# Hsp90 inhibition differentially destabilises MAP kinase and TGF-beta signalling components in cancer cells revealed by kinase-targeted chemoproteomics

**DOI:** 10.1186/1471-2407-12-38

**Published:** 2012-01-25

**Authors:** Armin Haupt, Gerard Joberty, Marcus Bantscheff, Holger Fröhlich, Henning Stehr, Michal R Schweiger, Axel Fischer, Martin Kerick, Stefan T Boerno, Andreas Dahl, Michael Lappe, Hans Lehrach, Cayetano Gonzalez, Gerard Drewes, Bodo MH Lange

**Affiliations:** 1 Department of Vertebrate Genomics, Max-Planck Institute for Molecular Genetics, Ihnestrasse 73, 14195 Berlin, Germany; 2 Cellzome AG, Meyerhofstrasse 1, 69117 Heidelberg, Germany; 3 International Center for Information Technology, University of Bonn, 53113 Bonn, Germany; 4 Cell Division Group, IRB-Barcelona, PCB, c/Baldiri Reixac 10-12, 08028 Barcelona, Spain; 5 Institucio Catalana de Recerca i Estudis Avançats, Passeig Lluís Companys 23 08010 Barcelona, Spain

## Abstract

**Background:**

The heat shock protein 90 (Hsp90) is required for the stability of many signalling kinases. As a target for cancer therapy it allows the simultaneous inhibition of several signalling pathways. However, its inhibition in healthy cells could also lead to severe side effects. This is the first comprehensive analysis of the response to Hsp90 inhibition at the kinome level.

**Methods:**

We quantitatively profiled the effects of Hsp90 inhibition by geldanamycin on the kinome of one primary (Hs68) and three tumour cell lines (SW480, U2OS, A549) by affinity proteomics based on immobilized broad spectrum kinase inhibitors ("kinobeads"). To identify affected pathways we used the KEGG (Kyoto Encyclopedia of Genes and Genomes) pathway classification. We combined Hsp90 and proteasome inhibition to identify Hsp90 substrates in Hs68 and SW480 cells. The mutational status of kinases from the used cell lines was determined using next-generation sequencing. A mutation of Hsp90 candidate client RIPK2 was mapped onto its structure.

**Results:**

We measured relative abundances of > 140 protein kinases from the four cell lines in response to geldanamycin treatment and identified many new potential Hsp90 substrates. These kinases represent diverse families and cellular functions, with a strong representation of pathways involved in tumour progression like the BMP, MAPK and TGF-beta signalling cascades. Co-treatment with the proteasome inhibitor MG132 enabled us to classify 64 kinases as true Hsp90 clients. Finally, mutations in 7 kinases correlate with an altered response to Hsp90 inhibition. Structural modelling of the candidate client RIPK2 suggests an impact of the mutation on a proposed Hsp90 binding domain.

**Conclusions:**

We propose a high confidence list of Hsp90 kinase clients, which provides new opportunities for targeted and combinatorial cancer treatment and diagnostic applications.

## Background

Hsp90 is part of the molecular chaperones family responsible for ATP-dependent folding and activation of proteins. Unlike other chaperones, Hsp90 is in most cases not involved in the *de novo *folding of proteins but stabilizes folded conformations and regulates protein degradation [1]. Another important difference is the selectivity of Hsp90 for its substrates, which therefore are called clients. Most of these client proteins are involved in signal transduction, including kinases, nuclear hormone receptors and transcription factors [2].

A potential role for Hsp90 in tumourigenesis has been suggested. Many types of tumours show an elevated level of Hsp90 correlated with a poor prognosis (e.g. [3,4]). This elevation is attributed to increased cellular stress due to tumour microenvironment, oncogenesis and increased dependency of mutated proteins on Hsp90 [1]. Known oncogenic Hsp90 clients include kinases such as SRC, CDK4, BRAF and ErbB2. Currently 14 Hsp90 inhibitors are evaluated in different stages of clinical development [5,6]. These compounds bind to the N-terminal ATPase-pocket, thereby disturb the chaperone cycle and lead to the depletion of Hsp90 substrates by proteasomal degradation [5]. Tumours are more susceptible to Hsp90 inhibitors than normal tissues because all Hsp90 protein is thought to become associated with its substrates in large chaperone complexes with high affinity for inhibitors like geldanamycin or 17-AAG [7]. To optimize future chemotherapeutic treatment and outcome, and to minimize side effects, it is essential to understand the molecular consequences of inhibiting Hsp90-dependent pathways and to define Hsp90-client protein interactions.

In the past, mass spectrometry (MS)-based proteomic approaches, yeast-two-hybrid screens and a genomic screen of yeast deletion strains were used to elucidate substrates and co-chaperones of Hsp90 and cellular pathways it acts upon [8-15].

This study represents the first comprehensive mapping of the Hsp90 client kinome. We employed a kinase-directed chemoproteomics approach [16] to assess the protein levels of kinases after Hsp90 inhibition by geldanamycin in frequently used cell lines of three different tumour origins and one primary cell line in order to identify novel clients of Hsp90 and define cancer-relevant differences between non-transformed cells and cancer cells.

## Methods

### Cell culture

Hs68, U2OS, SW480 and A549 cells were obtained from American Type Culture Collection (ATCC) and grown in DMEM supplemented with 10% FCS and 1% L-Glutamine at 37°C and 5% CO_2_. Glucose content was 1 g/l for U2OS and 4 g/l for all other cell lines. Cells were treated with 1.78 μM (1 μg/ml) geldanamycin (Biomol) solubilised in DMSO (Merck) for 12 or 24 h or with DMSO alone (control) for 24 h. Experiments were performed twice, in an independent manner. Cells were lysed in 50 mM Tris pH 7.4, 5% glycerol, 1.5 mM MgCl_2_, 150 mM NaCl, 1 mM Na_3_VO_4_, 25 mM NaF, 0.4% NP-40 and 1 mM DTT. For combined inhibition of the proteasome and Hsp90 20 μM of MG132 (Sigma) or the same volume of DMSO as a control were added for the last 6 h of geldanamycin treatment.

### Mass spectrometry

Lysates were used for kinobeads analysis as previously described [16]. Experimental design, mass spectrometry and statistical analyses are detailed in the Additional file 1. Only kinases with a *P*-value < 0.05 between duplicates were considered for further analysis.

### Next generation sequencing

Agilent Sure Select Enrichment of exonic regions, SOLiD next generation sequencing and bioinformatic workflow are detailed in Additional file 1.

### Structural analysis

Structural information on ErbB1/EGFR (PDB 1m14) was obtained from the Protein Data Bank (PDB) and the structure of RIPK2 is from the SWISS-MODEL Repository [17], based on template 2eva from PDB. Visualizations were done with PyMol.

## Results

### Targeted proteomics quantifies relative changes of kinase levels after Hsp90 inhibition

Hsp90 is required for the function and stability of a multitude of oncogenes. To better understand the complexity of Hsp90-dependent cellular signalling in normal and in cancer cells we analyzed the effect of the Hsp90 inhibitor geldanamycin on kinase abundance at 12 and 24 h. Kinases, which constitute the largest group of Hsp90 clients, are often difficult to quantify in whole cell protein extracts by mass spectrometry (MS) due to their low abundance, which hampers their quantitative detection in large numbers. In order to circumvent this problem, we enriched kinases using a sepharose matrix with immobilized broad spectrum kinase inhibitors (kinobeads) prior to quantitative MS analysis [16,18]. This technology allows the precise differential quantification of kinase expression levels. Experiments were carried out as independent biological replicates in order to ensure reproducibility and reliability.

**Figure 1:**
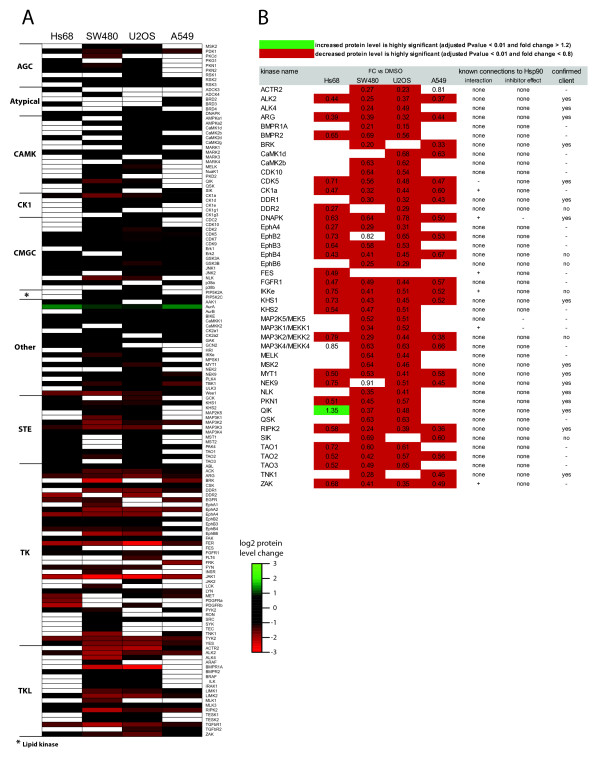
**Changes in kinase abundance upon geldanamycin treatment**. Relative quantification of kinases identified from Hs68, SW480, U2OS and A549 cells treated with geldanamycin for 24 h following enrichment on Kinobeads (from two independent experiments). Fold change refers to the ratio between geldanamycin-treated and DMSO-treated cells. **a**) Heat map representation of the effect of Hsp90 inhibition on the level of the 144 quantified kinases (log2 scale). Kinases are grouped in classes according to structural similarity of their catalytic domain. **b**) List of putative new Hsp90 kinase clients identified. Kinases were selected as client candidates if their log2 ratio (fold change treated versus untreated cells) is greater than-0.5 in Hs68 cells or in at least two cancer cell lines. The results are compared to literature data for evidence of previous associations (Additional file 5).

As a starting point we used the Hs68 primary foreskin fibroblast cell line to analyze the effects of geldanamycin on kinase levels in non-transformed cells. These results were compared to the response to the same treatment on cancer cell lines of different origin: osteosarcoma (U2OS), colon adenocarcinoma (SW480) and lung adenocarcinoma (A549). Upon kinobeads enrichment we quantified a total of 144 kinases from those four cell lines using a combination of isobaric mass tags and targeted MS technology [19] (Figure 1a and Additional file 2: Table S1). Among these, 46 have been described before as likely Hsp90 client proteins (Additional file 3: Table S2). We confirmed a significant decrease in the level of all of these kinases in at least one cell line after 24 h of geldanamycin treatment. More surprisingly, 26 of the 29 known Hsp90 client kinases that we also quantified in the reference Hs68 cell line showed significantly reduced levels after 24 h of treatment, including well known cancer-relevant proteins like EGFR, Met and PDGFRα/β. This was unexpected since healthy cells are thought to be poorly responsive to geldanamycin treatment. Alternatively, Hs68 cells may not be a perfect substitute for a normal, healthy cell, therefore minimizing the difference to cancer tumoural cells. Overall, 69% of all kinases quantified from Hs68 cells display significantly reduced levels after 24 h, a figure substantially lower than the 80 to 88% observed in the three cancer cell lines. Increased kinase abundance upon geldanamycin treatment was detected for nine kinases from Hs68 cells including JNK2, p38a, RSK2, ERK2 and AMPKA1, but only Aurora A kinase exhibited an increase in excess of 50% (+155%). Only Aurora A kinase is more abundant in kinobeads precipitates from the three cancer cell lines (from +85% in U2OS to +160% in A549 cells). The expression level of Aurora A is strongly regulated during cell cycle with a peak in mitosis. It is possible that the observed increase is due to an arrest of the cells at the G2/M checkpoint or in mitosis. Changes in relative kinase abundances were confirmed by immunoblotting (Additional file 4: Figure S1). As an internal control of geldanamycin treatment, components of the Hsp90 chaperone machinery are, as expected, strongly up-regulated when quantified directly from lysate (Additional file 3: Table S3) [20].

### Identification of potential new Hsp90 clients

We sought to identify potential new Hsp90 client proteins from our dataset to expand the knowledge about targets of Hsp90-based therapy. As classification criteria for new Hsp90 clients we set a protein level decrease after 24 h geldanamycin treatment of at least 30% (*P*-value < 0.01%) when compared to untreated cells, in either Hs68 or in at least two cancer cell lines (high confidence) or in only one cancer cell line. We retrieved 44 high confidence candidate clients (Figure 1b and Additional file 3: Table S2) with an average protein level reduction of 48%. We find the same extend of decrease for several known clients, which suggests a high probability that these are true clients. Amongst the high confidence candidates we identified five Ephrin receptors, MAP2K5, three MAP3Ks, all three Tao kinases, CK1a, CDK5, PLK4, NEK9, MYT1, DDR1 and DDR2, which points to a broader involvement of Hsp90 in signalling processes than previously thought. A few of these kinases (e.g. MAP3K1, CK1a, FES, IKKe) have already been shown to interact with Hsp90, but we found no evidence of Hsp90 inhibition effects on these kinases described in the literature (Figure 1b). The decrease of the levels of BMP receptors (BMPR1a, BMPR2, ALK2, and ACTR2) implies the Hsp90 machinery in BMP signalling. The BMP pathway has been proposed to have a role in cancer progression similar to that of TGF-beta signalling [21,22].

### Changes in kinase levels upon Hsp90 inhibition are rapid and dynamic

**Figure 2:**
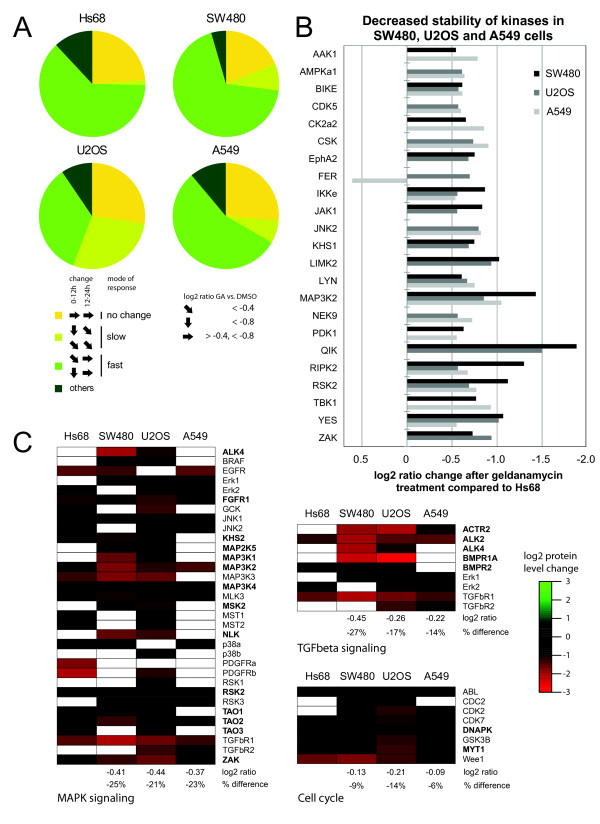
**Differential effects of Hsp90 inhibition in primary cells and in cancer cells lines**. **a**) Quantitative mapping of kinase populations in Hs68, U2OS, SW480 and A549 cells after geldanamycin treatment at two time intervals. Protein level changes over time at 12 h and 24 h were analyzed and kinases with similar behaviour were grouped as defined in the main text. Pie charts show for each cell line the relative proportion of respective groups compared to all quantified kinases. Log2 ratio fc (treated versus untreated cells) of -0.4,-0.8 and 0.4 were used as thresholds. **b**) Many kinases are strongly decreased in cancer cells when compared to non-transformed Hs68 cells. Kinase log2 ratio fc (24 h treated versus untreated cells) of cancer cells are compared to those of Hs68 cells. Differences of more than -0.5 were considered as differential and the graph shows kinases with an effect in at least two cell lines. **c**) Kinases from heat map data from Figure 1A are grouped according to KEGG (Kyoto Encyclopedia of Genes and Genomes) pathway classification. Candidate Hsp90 clients are in bold. Delta average of the log2 ratio (24 h treated versus untreated) and the respective percentage difference between each cancer cell line and Hs68 is displayed. fc - fold change.

To gain insight into the temporal dynamics of kinase level changes we investigated geldanamycin effects after 12 h and 24 h treatment. We classified kinases into four groups according to their pattern of protein level change (Figure 2a and Additional file 3: Table S4). The first class includes kinases that have unchanged levels following drug treatment. The second group displays decreased levels at 12 h, but no further decrease at 24 h (Figure 2a, "fast" decrease). The third group shows reduced levels at 12 h and further decrease at 24 h (Figure 2a, "slow" decrease). The last class regroups kinases presenting other patterns. In Hs68 cells we observe that 63% of all kinases show "fast" decrease kinetics, 1% a "slow" decrease and 24% appear unaffected. Amongst cancer cell lines, SW480 cells showed the largest similarity to Hs68 with more kinases with either "fast" (68%) or "slow" (8%) decrease kinetics, while less are unaffected (19%). U2OS cells differ prominently from Hs68 cells by an increased number of kinases with "slow" decrease kinetics (28%) along with a reduction of "fast" decrease kinetics kinases of the same magnitude. A549 cells display an intermediary pattern between those of Hs68 and SW480 cells. Therefore, some differences can be observed between the reference cell line and tumour cells, but no striking discrepancy in the kinetics of kinase amount reduction.

### Cellular pathways are differentially impacted by Hsp90 inhibition

Knowledge about the client response to Hsp90 inhibition is often derived from cancer cells. However, this might not reflect the behaviour of Hsp90-client interactions in normal, healthy cells. The distinction is of particular interest to predict possible side effects in therapy. Recently, it has been shown that Hsp90 inhibition indirectly promotes the growth of metastasing prostate carcinoma cells in the bone by primarily affecting the normal tumour-surrounding tissue [23].

Even though expression of many kinases is decreased in the primary cell line Hs68 following geldanamycin treatment, the level of some kinases appears unaffected by the treatment, or is slightly increased in Hs68 despite a strong decrease in cancer cells (e.g. QIK, BIKE, RSK2). It is possible that cancer cells show more generally a stronger decrease of key kinases than that observed in Hs68 cells after treatment. Hence, we compared protein level changes following geldanamycin treatment (24 h) of the 75 kinases quantified in Hs68 with those of cancer cells. We assumed a strong differential response to geldanamycin treatment when the difference in the log2 of the fold change (treated versus untreated) between a cancer cell line and Hs68 was greater than -0.5 (corresponding to a difference of about 30%). This threshold was met in at least one cancer cell line by 39 (52%) kinases (Additional file 3: Table S5) and by 23 kinases in at least two cell lines, including CDK5, CSK, LYN, RSK2 and YES (Figure 2b). Indeed, this points towards an increased responsiveness of cancer cell lines. We suggest that this reflects their stronger dependency on Hsp90, which has been connected to a higher affinity to inhibitors [7].

Looking on a pathway level using the KEGG classification we found the MAPK signalling pathway as the most prominent pathway in our dataset with a total of 33 kinases including 14 candidate clients (Figure 2c)[24]. For TGF-beta signalling, 9 kinases were identified, with five selected as putative new Hsp90 clients (ACTR2, ALK2, ALK4, BMPR1A and BMPR2). We also identified 8 cell cycle related proteins. To assess the overall impact of geldanamycin treatment on these pathways, we compared the average protein level change (treated versus untreated) for the non-transformed Hs68 cell line with that of each cancer cell line (only kinases quantified in the two cell lines being compared have been considered). In all three cases, kinases of the MAPK pathway were significantly less affected in the Hs68 cell line. The reduction of average kinase abundance for cancer cell lines as compared to Hs68 was 25% (SW480), 21% (U2OS) and 23% (A549). Similar results were obtained for the TGF-beta pathway with cancer cell lines exhibiting more pronounced effects (between 14 and 27%). Strikingly, however, when kinases involved in cell cycle regulation were considered, the difference between Hs68 and the three other cell lines was small (Figure 2c). This indicates that pathways may be differentially affected by Hsp90 inhibition in primary and cancer cells.

### Kinase dependency on Hsp90 is drastically increased in cancer cells

Geldanamycin treatment impacts the protein levels of a considerable fraction of the proteome either in a direct, Hsp90-dependent manner (for client proteins) or by indirect mechanisms [25]. Therefore, we determined to what extent the observed downregulation effects were a consequence of Hsp90-mediated proteasomal degradation by combining geldanamycin treatment with proteasome inhibition by MG132 during the last 6 hours. We choose to compare Hs68 and SW480 cells to detect potential differences between a primary and a cancer cell line. 53 and 91 kinases were identified and quantified from Hs68 and SW480 cells, respectively (Additional file 6: Table S6). After geldanamycin treatment, 40 kinases (75%) from Hs68 cells and 80 kinases (88%) from SW480 cells were recovered at significantly lower levels as compared to untreated cells (Additional file 6: Table S6). The amplitude of the decrease of kinase levels is larger than for the initial experiment and much bigger from SW480 than from Hs68 lysates: 50 kinases with levels reduced more than 75% in SW480 versus 10 in Hs68 (respectively 63% and 25% of all decreased kinases), likely due to a more efficient geldanamycin treatment (see Figure 2b). The list of kinases exhibiting the most pronounced downregulation closely matches the results of the first set of experiments. Additionally we identified kinases strongly decreased by geldanamycin that were not quantified in the previous experiment: STK33 and CamK2γ in SW480 and ACTR2 and Eph4α in Hs68 cells.

**Figure 3:**
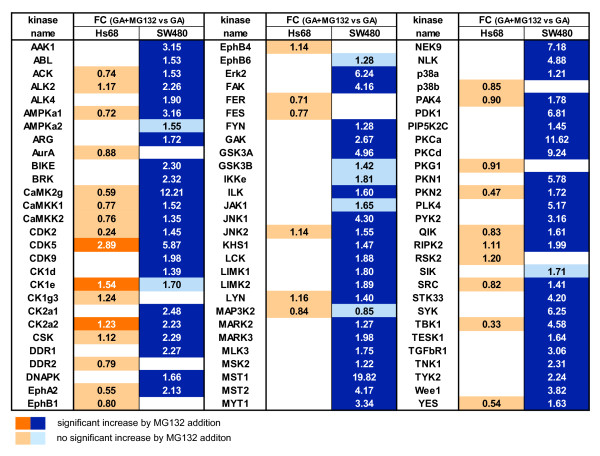
**Proteasome inhibition effects on kinase levels in geldanamycin-treated cells**. Relative quantification of kinases identified from Hs68 and SW480 cells treated with geldanamycin for 24 h with or without MG132 addition following enrichment on Kinobeads (from two independent experiments). Fold change refers to the ratio between geldanamycin + MG132-treated and geldanamycin-treated cells. Kinases are highly significantly increased (dark orange or blue) when fold change is > 1.2 and *p*-value < 0.01. FC - fold change.

The addition of the proteasome inhibitor MG132 in the last 6 h of geldanamycin treatment was expected to stop degradation of client kinases and therefore increase their levels [5]. Non-client kinases which may exhibit downregulation downstream of client kinases or are affected via another mechanism following geldanamycin addition should not show this trend. In SW480 cells, 64 (86%) of the kinases affected by geldanamycin treatment showed increased levels following addition of MG132 (Figure 3). Some kinases, which displayed a reduction of more than 75%, again reached levels similar to those detected in untreated cells, e.g. MST1, MST2, CamK2γ or PRKCα. Strikingly, only three kinases downregulated after geldanamycin treatment from Hs68 cells, CDK5, CK2a2 and CK1e, showed increased levels following MG132 addition (Figure 3). This result suggests that the observed decrease of kinase levels in Hs68 is not due to strong Hsp90-dependent proteasomal protein processing.

In this dataset we quantified 28 known client kinases of which 25 showed the expected upregulation of their protein levels after MG132 addition, validating our approach to identify Hsp90 kinase clients. Among the proposed 44 clients (Figure 1b) 16 out of the 23 new clients quantified in the MG132 experiment were found significantly increased after combined geldanamycin and proteasome inhibitor treatments (fold change geldanamycin + MG132 vs. geldanamycin > 1.2 and *p*-value < 0.01), supporting the notion that these are true Hsp90 clients. Additionally we find 18 kinases among our good confidence candidate group confirmed by this method (Additional file 3: Table S2). In total we identified 64 kinases from Hs68 and SW480 cells which can be classified as true Hsp90 clients, because their degradation by geldanamycin was significantly rescued by MG132 treatment. Notably, kinases from SW480 cells were more dependent on Hsp90 than their counterparts in Hs68 cells.

### Structural analysis of kinase mutations and differential inhibition effects

Mutations can modify the dependency of oncogene proteins for Hsp90 chaperoning. For example, normal c-Src requires Hsp90 only at an early stage for maturation. Constitutive kinase activity of the unstable truncated mutant requires a stronger association, rendering it more susceptible to Hsp90 inhibition [1]. In contrast, stabilising mutations can render PLK1 more stable, less dependent of Hsp90 activity and hence less susceptible to degradation upon Hsp90 inhibition [26].

**Figure 4:**
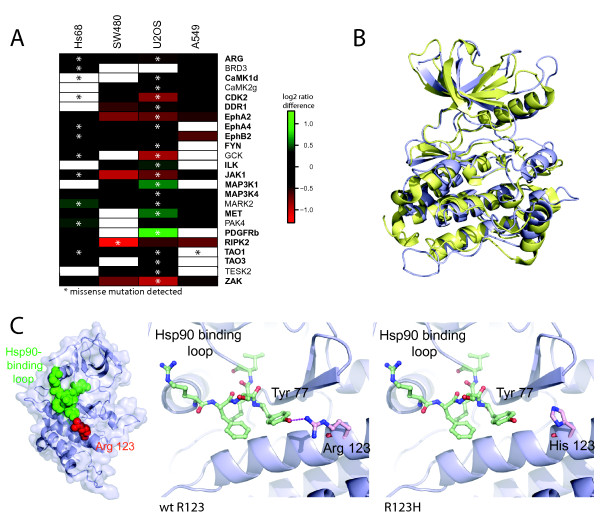
**Analysis of the effect of mutations on the response to Hsp90 inhibition**. **a**) Kinases with a mutation detected by next generation sequencing. Shown are the differences in the response to geldanamycin. For each kinase the non-mutated cell line most weakly responding was used as a reference for comparison with the response in the other cell lines. Kinase names printed in bold are known or newly identified candidate clients of Hsp90. **b**) Superposition of the kinase domains of ErbB1/EGFR (yellow), and RIPK2 (blue) shows structural conservation. **c**) Potential mechanism for the differential response of RIPK2 to geldanamycin. Overview of kinase domain (left), close-up of affected region in wild type (middle) and mutant (right) protein. In wild type RIPK2 R123 (red) interacts with Tyr77 of the putative Hsp90 recognition loop (green) (left, middle panel). In the R123H mutant this interaction is lost (right panel).

We examined by next generation sequencing the coding regions of all 144 kinase genes that we had characterised by targeted proteomics in Hs68, SW480, U2OS and A549 cells. Among these, 73 had at least one missense mutation (Additional file 7: Table S7). We filtered out mutations that were not present in dbSNP and that were not predicted to affect protein function by using Polyphen and MutationTaster (Additional file 1). After filtering we obtained a set of 24 kinases (Figure 4a).

In order to test if there is a correlation between the protein level after geldanamycin treatment and the presence of a mutation, we compared the protein level changes in wt and mutated cell lines (Figure 4a). Indeed, we find the strongest differential response to geldanamycin for seven kinases in the cell line carrying the mutation (Cdk2, Ilk, MAP3K1, MET, PAK4, PDGFRb, RIPK2). Among these, five kinases show an elevated protein level after geldanamycin treatment compared to a cell line lacking the mutation. These are potentially examples of clients that have, at least partially, lost their dependency on Hsp90 and evade Hsp90 inhibition effects, which could impair effectiveness of cancer treatment. We also identify kinases for which the same mutation leads to a stronger decrease after geldanamycin treatment in only one of two different cell lines (e.g. JAK1), while the response in the second one is unaltered. These mutations are likely independent of Hsp90 or at least depend on additional factors that are outside the scope of this work (Figure 4a). In many other cases, the presence of a mutation shows limited impact on kinase levels (e.g. TAO1, EphA4, FYN, ARG). These mutations do not affect the stability of the kinases in such a way that it modifies their dependency on Hsp90 chaperone machinery.

We wanted to investigate the possible molecular basis of the effects we observed. Therefore we used existing structural information for the kinase which was most strongly affected by a mutation, receptor-interacting serine-threonine kinase 2 (RIPK2). We mapped the affected residues and tried to predict the functional impact of the mutation located within the kinase domain. A charge-rich loop of the kinase domain has been proposed as the binding region of Hsp90 on some clients like ERBB1/EGFR [27]. We found structural conservation between ERBB1 and RIPK2, which enabled us to map this loop onto the structures of our candidate protein (Figure 4b). The residue R123 in RIPK2 is adjacent to the charge-rich loop and directly interacts with Tyr77 within this region (Figure 4c left and middle). In the R123H mutant this interaction is lost, which likely affects the geometry of the Hsp90 recognition loop (Figure 4c right). This in turn could require a stronger association with Hsp90 in order to maintain the tertiary structure. This idea is supported by the finding that RIPK2 is an Hsp90 client only in SW480 cells, but not in Hs68 cells (Figure 3). We analysed the mutational pattern of all kinases quantified in our Kinobead assay and could correlate a subset of these mutations with a differential response of the kinases to Hsp90 inhibition. In the case of RIPK2 we propose that the mutation affects kinase stability by changing the geometry of the putative Hsp90 recognition site in this protein.

## Discussion

In the past, several studies have analysed the cellular response to multiple Hsp90 inhibitors in different cell lines and organisms. Genome-wide expression profiling has been employed to monitor the cellular response to Hsp90 inhibition and revealed affected genes and markers for monitoring of effective Hsp90 inhibition in clinical treatment like Hsp70 [28]. However, these transcriptional events are indirect as Hsp90 regulates mostly the protein stability of its clients. Some studies have however used proteomics to monitor protein levels and revealed affected pathways like for example chromatin remodelling or MAPK, WNT, NF-kB and TGF-beta signalling [25,29]. However kinases, the largest group of clients, were underrepresented in these studies. To close this gap we focused on the analysis of the response to Hsp90 inhibition of a large number of kinases by measuring their protein level changes. We compared the rate of down- or upregulation of our kinase-focussed assay with the two abovementioned previous studies that probed the unfiltered proteome. Schumacher *et al*. identified 111 differentially expressed proteins of which about 41% were overexpressed and 59% underexpressed [25]. Similar values were found by Maloney *et al*. (26 proteins in total, 46% over- and 54% underexpressed) [29]. Comparing these values to significant downregulation of 69% to 88% of all identified kinases by our assay argues for a high specificity of kinase level reduction after Hsp90 inhibition.

A higher affinity to Hsp90 inhibitors has been reported in cancer cells [7]. We investigated if there were different kinetics of client degradation between normal and cancer cells in response to geldanamycin. Interestingly, we did not find striking differences arguing that kinetic properties do not contribute to the increased response to Hsp90 inhibition in cancer cells. We only observed slower kinetics in U2OS cells, which may be due to reduced transport of the drug into the cell or differences in metabolism of the drug. For example, it has been shown for 17-allylamino,17-demethoxygeldanamycin (17-AAG), a geldanamycin derivative, that activity of the quinone reductase DT-diaphorase (gene name: NQO1) is positively correlated with the growth-inhibitory activity of 17-AAG, because DT-diaphorase converts 17-AAG into a form more potent for Hsp90 inhibition [30].

Our kinase-enrichment approach allowed us to monitor the effect of the Hsp90 inhibitor geldanamycin on 144 kinases. From this data we were able to identify 44 high confidence client kinase candidates (Figure 1b) with a strong representation of MAPK and TGF-beta signalling components (Figure 2c). The additional treatment with proteasome inhibitor MG132 allowed us to discriminate between Hsp90 client candidates that likely undergo ubiquitination and proteasomal degradation (their cellular levels are at least partially recovered in presence of MG132) and kinases that are affected by geldanamycin treatment more indirectly - for example via transcriptional regulation if downstream of a client kinase - or via another mechanism (their cellular levels should not be significantly changed in presence of MG132). Whereas a majority of kinases affected by geldanamycin treatment appears to be true client Hsp90 proteins in SW480, only few kinases displays a similar behaviour in Hs68. Based on these results, we propose a list of Hsp90 client kinases (Figure 3). This list regroups 64 kinases and includes many tyrosine kinases (e.g. Fyn, Lyn, Src, Yes, Abl, Arg, Tyk2) or tyrosine-like kinases (ALK2, ALK4, RIPK2, ILK, TGFBR1, MLK3), two phylogenic branches of kinases that have previously been shown to include most of the Hsp90 kinase clients [27]. Ephrin receptors distinguish themselves among tyrosine kinases since - with the noticeable exception of Ephα2 -, they do not appear to be Hsp90 clients, at least in SW480 cells. Among the Serine/Threonine kinase groups, we find well-described Hsp90 clients like CDK2, CDK9, CK2α1, CK2α2 or TBK1 but also some kinases that were thought not to be Hsp90 clients based on their sequence [27] like CDK5, PKCα, PKCβ or MAPK1/ERK2. Conversely, among kinases that did not appear as Hsp90 clients in this study, we find some kinases that have been previously described as putative Hsp90 clients like GSK3β, JAK1 or FER. Our list of Hsp90 client kinases is therefore significantly different from those that have been proposed so far [27,31]. Geldanamycin treatment affects many pathways and has major impact on the entire proteome [25]. It is therefore not too surprising that the level of many kinases is significantly modified even if their levels are poorly regulated by Hsp90 machinery, like many kinases from Hs68 cells. This data strongly support the hypothesis that the role of Hsp90 as a kinase chaperone is much less preeminent in healthy primary cells than in a cancer cell type as colon adenocarcinoma SW480 cells. We conclude from our results that in normal cells the majority of downregulated kinases following geldanamycin treatment is driven by indirect, non Hsp90-dependent mechanisms of degradation. In contrast, in cancer cells the majority of kinases appear to be dependent on Hsp90 chaperoning.

Many Hsp90 inhibitors have been developed and several are presently undergoing clinical evaluation [5]. This is the first study that focuses on the impact of Hsp90 inhibition on a broad spectrum of the kinome. Our results reveal an impact of Hsp90 inhibitors on more wide-ranging types of kinases, and hence pathways, than previously thought (Figure 1b and 2c). This is of special clinical interest for the inhibition of feedback loops that often arise in single targeted therapy and that have been acknowledged as a resistance mechanism and escape route for cancers to evade treatment. For example the use of mTOR inhibitors leads to the PI3K-dependent activation of MAPK and Akt signalling, which both are targeted by Hsp90 inhibitors [32,33]. Our discovery of many new Hsp90 targets further supports the use of Hsp90 inhibitors in combinatorial treatment, especially as a means to suppress feedback loops, because it affects even more processes than anticipated so far.

Many kinases annotated by KEGG as members of TGF-beta signalling identified in our study belong to the bone morphogenetic protein (BMP) signalling pathway. We show for the first time that BMP receptors (BMPR1A, BMPR2, ACTR2, ALK2 and ALK4) exhibit downregulation after Hsp90 inhibition (Figure 1). BMP proteins are members of the TGF-beta superfamily and have important functions including embryonic development, bone formation and tissue homeostasis [34]. BMPs bind to BMP receptors that mediate signals mostly via phosphorylation of SMADs [34]. In cancer, this pathway has been linked for example to bone metastases formation in breast, prostate and lung cancer and control of cell proliferation. The outcome of BMP receptor signalling is strongly cell type- specific and also dependent on which BMPs are present [35]. Therefore the consequences of downregulation of multiple BMP receptors by Hsp90 inhibition could be diverse in different tissues. In prostate cancer BMPR1A and especially BMPR2 downregulation has been correlated with disease progression and activity of BMPR2 has been shown to function in a proliferation suppressive way [36,37]. Our findings suggest that the use of Hsp90 inhibitors in prostate cancer might, by the downregulation of BMPRs, lead to an unintended promotion of proliferation and metastasis formation, thereby counteracting or attenuating the beneficial effects exerted on other pathways and limiting its clinical use. In line with this, results from a clinical trial with hormone-refractory prostate cancer suggest that Hsp90 inhibitors are no effective agents when used in monotherapy [38]. If this is attributable to their effects on BMPRs remains to be determined. In contrast, in breast cancer BMPR1A activity was shown to promote cell proliferation via SMADs, whereas results for BMPR2 are contradictory [39-41]. This indicates that there might be a more promising therapeutic window for the use of Hsp90 inhibitors to reduce BMP signalling in certain breast cancers, in which setting several Hsp90 inhibitors are currently tested [5].

Our study identified many kinases of the JNK and p38 MAPK pathways, which are involved in diverse processes like for example stress response, inflammation, cell proliferation, survival and migration. Both pathways are often deregulated in cancer, however the often context-specific oncogenic and tumour suppressive functions impede the prediction of a pharmacological intervention [40]. We identified new Hsp90 targets within the p38, JNK and also Erk5 MAPK signalling cascades on the level of MAPKKKs (MEKK2/3/4, MLK1/3, TAO1/2 and ZAK) and upstream regulatory MAP4Ks (GCK, KHS1, KHS2) and could show an increased downregulation in cancer cells upon geldanamycin treatment for some of them (Figure 1 and 2b). Several inhibitors of p38alpha and JNK have been developed, but have side effects or lacked specificity [42]. We do not propose Hsp90 inhibitors as a single agent treatment in this setting, however it might prove useful for combinatorial treatment with future improved inhibitors against JNK and p38, because they can downregulate several upstream components of the MAPK cascades, likely increasing the efficacy of inhibition. As Hsp90 inhibitors act more specifically on tumours this additional effect would be limited to the target tissue, which likely minimises systemic side effects.

## Conclusions

Our findings emphasise the influence of the underlying genetic background of individual tumours on the response to Hsp90 inhibitors. In addition we find a large range of kinases more strongly destabilized and more dependent on Hsp90 chaperoning in the examined cancer cell lines when compared to a primary, phenotypically normal cell line. In the future the establishment of chemoproteomic and mutational profiles, that analyse responsiveness to Hsp90 inhibition in different cancer types, will help to identify patients that can benefit from this treatment especially by combinatorial treatment. Recently, efforts to establish mutational profiles of lung cancer have been undertaken and showed promising results for mutation-specific treatment, for example of tumour cells showing *ras *mutations, with an Hsp90 inhibitor [43].

## Competing interests

The authors declare that they have no competing interests.

## Authors' contributions

AH was involved in the experimental design, conducted the Hsp90 inhibition experiments, proteomics data interpretation, prepared the figures and prepared the manuscript.GJ performed the experimental design and coordination, proteomics data analysis and interpretation and prepared the manuscript. MB designed the mass spectrometry experiments and coordinated the mass spectrometry platform. HF developed Bioinformatic tools and performed the bioinformatic analysis of mass spectrometry data. HS performed the structural analysis of RIPK2 and prepared part of the related figure and manuscript parts. MRS conceived the next sequencing experiments, conducted the sequencing experiments and data analysis and contributed to the manuscript preparation. AF and MK performed the sequencing data analysis and bioinformatics analysis. STB and AD conducted wet lab and sequencing experiments for next generation sequencing. ML conceived and supervised the structural analysis. HL contributed to experimental design. CG envisioned the first part of the study, contributed to experimental design and manuscript preparation. GD developed the experimental design and supervised proteomics experiment execution, data interpretation and contributed to the manuscript preparation. BMHL envisioned the study, participated in its design, coordination and final manuscript preparation. All authors read and approved the final manuscript.

## Pre-publication history

The pre-publication history for this paper can be accessed here:

http://www.biomedcentral.com/1471-2407/12/38/prepub

## Supplementary Material

Additional file 1 **Supplementary Methods**. Detailed methods for experimental design, mass spectrometry, statistical analyses and next generation sequencing. Click here for file

Additional file 2 **Supplementary_Table 1**. Detailed results of the kinobeads-MS experiments with geldanamycin treatment. Click here for file

Additional file 3 **Supplementary_Table 2-5**. Table 2 summarizes quantification data and indicates known clients including references for interactions and Hsp90 inhibitor effects. Table 3 lists protein level changes of heat shock proteins after geldanamycin treatment for 24 h. Table 4 lists kinetics of individual kinases in all four cell lines. Table 5 differential quantification of kinases when compared to Hs68 following 24 h treatment with geldanamycin. Click here for file

Additional file 4 **Supplementary_Figure 1**. Shown are the western blot results of two experiments comparing DMSO and geldanamycin treatment for 24 h. Click here for file

Additional file 5 **Supplementary References**. Reference list for literature cited in Supplementary Table 2. Click here for file

Additional file 6 **Supplementary_Table_6**. Detailed results of the kinobeads-MS experiments with combined geldanamycin and MG132 treatment. Click here for file

Additional file 7 **Supplementary_Table_7**. Results of the sequencing of 144 kinase genes in Hs68, SW480, U2OS and A549 cells. Click here for file

Additional file 8 **Supplementary_Documents_Description**. Description of each supplementary table content and supplementary figure description. Click here for file
