# Supplementary material for: Hsp90 inhibition differentially destabilises MAP kinase and TGF-beta signalling components in cancer cells revealed by kinase-targeted chemoproteomics
Source: BMC Cancer. 2012 Jan 25;12:38. doi: 10.1186/1471-2407-12-38 (PMC3342885; doi:10.1186/1471-2407-12-38)
Supplement: Additional file 1 — Supplementary Methods. Detailed methods for experimental design, mass spectrometry, statistical analyses and next generation sequencing. [file 1471-2407-12-38-S1.DOC]

Supplementary Methods

Experimental design

Each experiment was performed as an independent duplicate. For each cell line, purifications were measured in MS as a group of six samples (Geldanamycin 24 hrs, Geldanamycin 12 hrs, DMSO 24 hrs (all experiment 1), Geldanamycin 24 hrs, Geldanamycin 12 hrs, DMSO 24 hrs (all experiment 2.) Captured proteins were trypsinized and each peptide mixture is tagged with a distinct isobaric tag. Tagged samples were pooled and analyzed by LC-MS/MS such that each peptide gives rise to six reporter signals in the MS/MS spectrum allowing precise quantification of each kinase

Sample preparation and LC-MS/MS analysis

Gels were cut into slices across the entire separation range and subjected to in-gel digestion. Peptide extracts were labeled with TMT™ (Thermo-Fisher Scientific) in 40 mM triethylammoniumbicarbonate (TEAB), pH 8.53. After quenching of the reaction with glycine labeled extracts were combined. Samples were dried *in vacuo* and re-suspended in 0.1 % formic acid in water and aliquots of the sample were injected into a nano-LC system (Eksigent 1D+) coupled to LTQ-Orbitrap mass spectrometers (Thermo-Finnigan). Peptides were separated on custom 50 cm x 75µM (ID) reversed phase columns (Reprosil) at 40°C. Gradient elution was performed from 2% acetonitrile to 40% acetonitrile in 0.1% formic acid over 4 hrs. The LTQ-Orbitrap was operated with XCalibur 2.0software. Intact peptides were detected in the Orbitrap at 30.000 resolution. Internal calibration was performed using the ion signal from (Si(CH_3_)_2_O)_6_H^+^ at m/z 445.120025. For CID up to 5000 ions (Orbitrap XL) were accumulated in the ion trap within a maximum ion accumulation time of 200 msec. For HCD target ion settings were 50000 (Orbitrap XL), respectively. Each survey spectrum triggered fragmentation of up to six peptide ions with both CID (NCE 35%) and HCD (NCE 70%) using a targeted data acquisition approach [99].

*Peptide and protein identification*

Mascot^TM^ 2.0 (Matrix Science) was used for protein identification using 10 ppm mass tolerance for peptide precursors and 0.8 Da (CID) tolerance for fragment ions. Carbamidomethylation of cysteine residues and TMT modification of lysine residues were set as fixed modifications and S,T,Y phosphorylation, methionine oxidation, N-terminal acetylation of proteins and TMT modification of peptide N-termini were set as variable modifications. The search data base consisted of a customized version of the IPI protein sequence database combined with a decoy version of this database. We accepted protein identifications as follows: i) For single spectrum to sequence assignments, we required this assignment to be the best match *and* a minimum Mascot score of 31 *and* a 10x difference of this assignment over the next best assignment. Based on these criteria, the decoy search results indicated <1% false discovery rate (FDR); ii) For multiple spectrum to sequence assignments and using the same parameters, the decoy search results indicate <0.1% false discovery rate.

*Peptide and protein quantification*

Centroided TMT reporter ion signals were computed by the XCalibur software operating and extracted from MS data files using customized scripts. Only peptides unique for identified proteins were used for relative protein quantification. Reporter ion intensities were multiplied with the ion accumulation time yielding an area value proportional to the number of reporter ions present in the mass analyzer. Fold changes are reported based on reporter ion areas and were calculated using sum-based bootstrap algorithm. Fold changes were corrected for isotope purity as described and adjusted for interference caused by co-eluting nearly isobaric peaks as estimated by the s2i measure (Savitski et al. 2010.)

Statistical analysis of data

*Filtering step*

In the Kinobeads experiments, a protein was quantified if identified with at least 2 unique peptides. In the whole proteome study, a protein was quantified if at least 4 good quality spectra that could be assigned unambiguously to a unique peptide and therefore to a single protein were identified

*Normalization of data*

Raw summed ion peak areas of proteins are expected to be confounded by several influence factors, namely a protein specific factor (due to biological differences, but also due to systematic biases between gel bands) and an isobaric label specific factor (sample specific factor). These confounding factors were removed robustly via a median polish (Tukey 1977). The remaining residuals after the median polish decomposition of the raw summed ion peak areas form the normalized summed ion peak areas.

*Integrating data from replicate experiments*

Only quantified proteins that have been identified in both replicates have been considered. We performed a quantile normalization (Smyth 2004) on the assumption that there should not be any overall major difference between replicate experiments

*Differential protein expression*

After data normalization and integration we used the limma method (Boldstad et al. 2003) known from gene expression analysis to estimate protein fold changes and to assess their statistical significance. Briefly, the idea is to perform a moderated t-test between treated and control measurements for each protein:


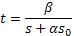


where ß is the fold change (log2 scale), s the standard deviation of the protein and s_0_ an estimate of the global spread of the data, which is weighted by a factor α estimated via the empirical Bayes method The fold change as well as the test statistic *t* are determined by a linear model fit. The limma method determines a p-value for each protein, which is adjusted for multiple testing via Benjamini-Hochberg’s procedure (Benjamini et al. 1995) controlling the false discovery rate.

*Implementation*

All analysis was conducted within the R statistical computing environment employing the R-packages *limma*, RODBC and reshape.

**Western Blotting:**

The experiment was carried out as described in the cell culture section, but in an independent manner. Kinases levels were analysed by western blotting using KPKS-1.2 protein kinase screen (Kinexus Bioinformatics Corp.) and samples were prepared according to company's instructions. Proteins of the extract were separated by SDS-PAGE and subsequently immuno-blotted. Up to three proteins per lane were labelled with antibodies on a 20 lane Immunetics Multiblotter. Antibody combinations were selected in a way to avoid cross reactivity. Only kinases identified in both experiments were used for comparison.

**Next generation sequencing and data analysis**

Agilent Sure Select Enrichment and SOLiD next generation sequencing

SOLiD library preparation of HS68, SW480, U20S and A549 cell line DNA was performed according to Agilent’s SureSelect Target Enrichment protocol for the Applied Biosystems SOLiD system. In brief, whole genomic DNA was sheared and end repaired. Sequencing adapters were ligated in a 30x excess. A size selection of DNA fragments between 150-200bp followed by a nick-translation and amplification step with Platinum polymerase (Invitrogen) and Pfu-Polymerase (Fermentas) was performed. The libraries were adjusted to 500ng in 3.4µl volume, added to the SureSelect Block solutions and hybridized for 24h at 65°C. Hybrids were extracted with 500ng M-280 streptavidin Dynabeads (Invitrogen) and finally eluted with 50µl Elution buffer. After amplification with Platinum polymerase the libraries were quantified by qPCR and DNA concentration was titrated to eventually yield 10-20% monoclonal templated beads in the emulsion PCR reaction using in total 1,6 billion P1-beads as input. Templated P1-beads were enriched and deposited (130 million beads per quarter slide (quad)) according to AB’s SOLiD bead enrichment and deposition protocol. Sequencing was performed with fragment sequencing chemistry.

Kinase structure study

Reads were aligned to HG19 using Applied Biosystem's Bioscope Alignment module in seed&extend and Single Nucleotide Variants were called by the Dibayes algorithm integrated in the Bioscope Package taking recommended parameters for targeted resequencing into account under medium stringency criteria.

SNP filtering workflow was initiated by mapping all variations against all transcript models generated by Ensembl (ensembl58.37, www.ensembl.org) leading to multiple annotations of one SNV for several loci. Sequencial filtering steps were performed to extract SNVs of interest: SNVs with a coverage greater than 10, within coding regions, not annotated in dbSNP131 with minor allele frequencies or average heterozygosity >0.01, leading to an amino exchange and to an impaired protein function using Polyphen and MutationTaster (http://genetics.bwh.harvard.edu/pph/ (Ref.93), http://neurocore.charite.de/MutationTaster/ (Ref. 94)).
