# Supplementary material for: Hsp90 inhibition differentially destabilises MAP kinase and TGF-beta signalling components in cancer cells revealed by kinase-targeted chemoproteomics
Source: BMC Cancer. 2012 Jan 25;12:38. doi: 10.1186/1471-2407-12-38 (PMC3342885; doi:10.1186/1471-2407-12-38)
Supplement: Additional file 4 — Supplementary_Figure 1. Shown are the western blot results of two experiments comparing DMSO and geldanamycin treatment for 24 h. [file 1471-2407-12-38-S4.PDF]

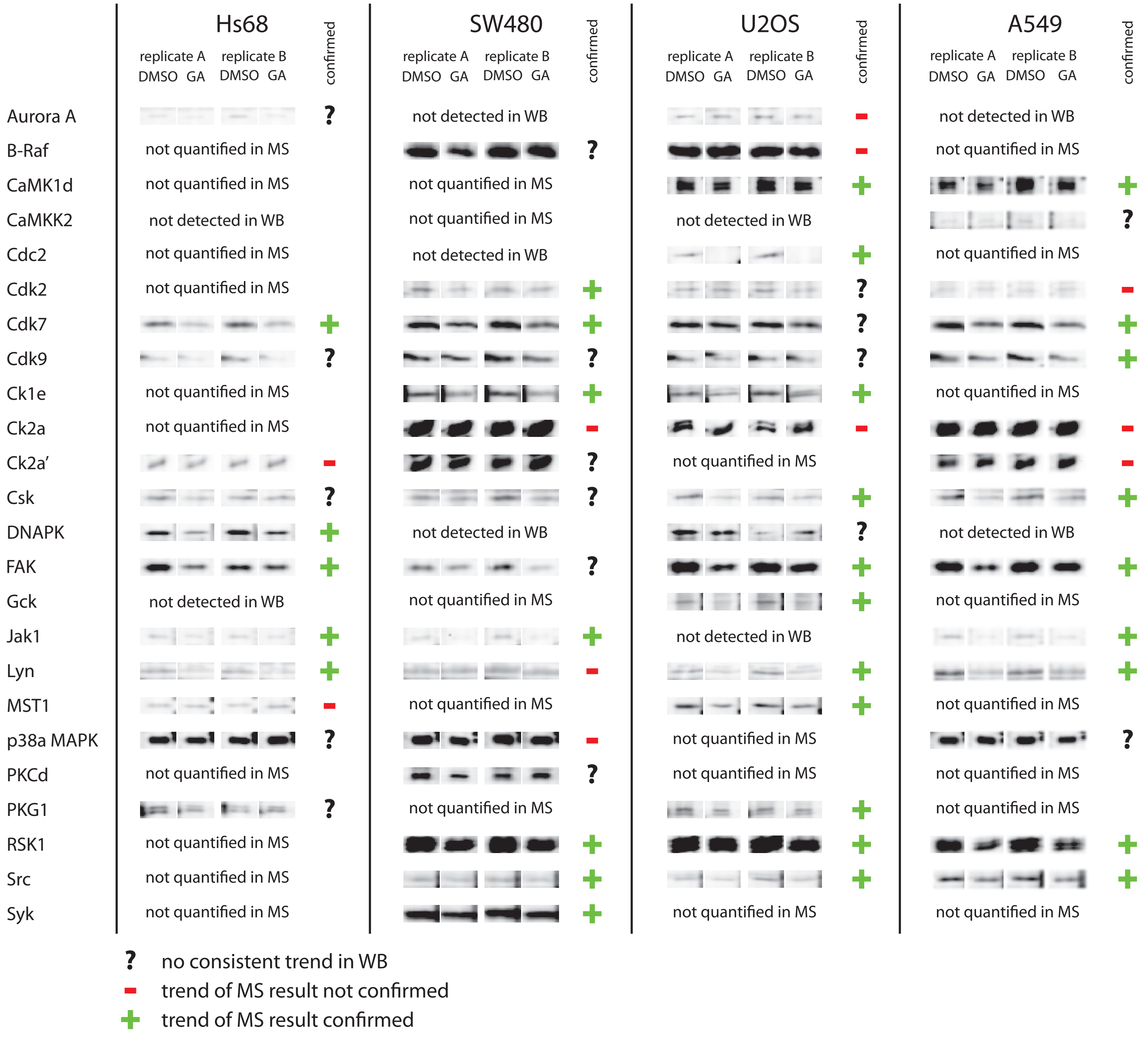


Supplementary Figure 1 - Haupt et al.
