# Supplementary material for: Hsp90 inhibition differentially destabilises MAP kinase and TGF-beta signalling components in cancer cells revealed by kinase-targeted chemoproteomics
Source: BMC Cancer. 2012 Jan 25;12:38. doi: 10.1186/1471-2407-12-38 (PMC3342885; doi:10.1186/1471-2407-12-38)
Supplement: Additional file 5 — Supplementary References. Reference list for literature cited in Supplementary Table 2. [file 1471-2407-12-38-S5.DOC]

Supplementary References:

1. Aligue, R., H. Akhavan-Niak, and P. Russell, *A role for Hsp90 in cell cycle control: Wee1 tyrosine kinase activity requires interaction with Hsp90.* EMBO J, 1994. **13**(24): p. 6099-106.

2. An, W.G., T.W. Schulte, and L.M. Neckers, *The heat shock protein 90 antagonist geldanamycin alters chaperone association with p210bcr-abl and v-src proteins before their degradation by the proteasome.* Cell Growth Differ, 2000. **11**(7): p. 355-60.

3. Annamalai, B., et al., *Hsp90 is an essential regulator of EphA2 receptor stability and signaling: implications for cancer cell migration and metastasis.* Mol Cancer Res, 2009. **7**(7): p. 1021-32.

4. Aoyagi, Y., N. Fujita, and T. Tsuruo, *Stabilization of integrin-linked kinase by binding to Hsp90.* Biochem Biophys Res Commun, 2005. **331**(4): p. 1061-8.

5. Banz, V.M., et al., *Hsp90 transcriptionally and post-translationally regulates the expression of NDRG1 and maintains the stability of its modifying kinase GSK3beta.* Biochim Biophys Acta, 2009. **1793**(10): p. 1597-603.

6. Basso, A.D., et al., *Akt forms an intracellular complex with heat shock protein 90 (Hsp90) and Cdc37 and is destabilized by inhibitors of Hsp90 function.* J Biol Chem, 2002. **277**(42): p. 39858-66.

7. Basso, A.D., et al., *Ansamycin antibiotics inhibit Akt activation and cyclin D expression in breast cancer cells that overexpress HER2.* Oncogene, 2002. **21**(8): p. 1159-66.

8. Bijlmakers, M.J. and M. Marsh, *Hsp90 is essential for the synthesis and subsequent membrane association, but not the maintenance, of the Src-kinase p56(lck).* Mol Biol Cell, 2000. **11**(5): p. 1585-95.

9. Bisht, K.S., et al., *Geldanamycin and 17-allylamino-17-demethoxygeldanamycin potentiate the in vitro and in vivo radiation response of cervical tumor cells via the heat shock protein 90-mediated intracellular signaling and cytotoxicity.* Cancer Res, 2003. **63**(24): p. 8984-95.

10. Bouwmeester, T., et al., *A physical and functional map of the human TNF-alpha/NF-kappa B signal transduction pathway.* Nat Cell Biol, 2004. **6**(2): p. 97-105.

11. Caldas-Lopes, E., et al., *Hsp90 inhibitor PU-H71, a multimodal inhibitor of malignancy, induces complete responses in triple-negative breast cancer models.* Proc Natl Acad Sci U S A, 2009. **106**(20): p. 8368-73.

12. Carter, T., et al., *A DNA-activated protein kinase from HeLa cell nuclei.* Mol Cell Biol, 1990. **10**(12): p. 6460-71.

13. Cissel, D.S. and M.A. Beaven, *Disruption of Raf-1/heat shock protein 90 complex and Raf signaling by dexamethasone in mast cells.* J Biol Chem, 2000. **275**(10): p. 7066-70.

14. Citri, A., et al., *Hsp90 recognizes a common surface on client kinases.* J Biol Chem, 2006. **281**(20): p. 14361-9.

15. da Rocha Dias, S., et al., *Activated B-RAF is an Hsp90 client protein that is targeted by the anticancer drug 17-allylamino-17-demethoxygeldanamycin.* Cancer Res, 2005. **65**(23): p. 10686-91.

16. De Nardo, D., et al., *A central role for the Hsp90.Cdc37 molecular chaperone module in interleukin-1 receptor-associated-kinase-dependent signaling by toll-like receptors.* J Biol Chem, 2005. **280**(11): p. 9813-22.

17. Dewaele, B., et al., *Activity of dasatinib, a dual SRC/ABL kinase inhibitor, and IPI-504, a heat shock protein 90 inhibitor, against gastrointestinal stromal tumor-associated PDGFRAD842V mutation.* Clin Cancer Res, 2008. **14**(18): p. 5749-58.

18. Donze, O. and D. Picard, *Hsp90 binds and regulates Gcn2, the ligand-inducible kinase of the alpha subunit of eukaryotic translation initiation factor 2 [corrected].* Mol Cell Biol, 1999. **19**(12): p. 8422-32.

19. Doong, H., et al., *CAIR-1/BAG-3 abrogates heat shock protein-70 chaperone complex-mediated protein degradation: accumulation of poly-ubiquitinated Hsp90 client proteins.* J Biol Chem, 2003. **278**(31): p. 28490-500.

20. Dote, H., et al., *Inhibition of hsp90 compromises the DNA damage response to radiation.* Cancer Res, 2006. **66**(18): p. 9211-20.

21. Falsone, S.F., et al., *A proteomic snapshot of the human heat shock protein 90 interactome.* FEBS Lett, 2005. **579**(28): p. 6350-4.

22. Fang, S., et al., *Heat shock protein 90 regulates the stability of MEKK3 in HEK293 cells.* Cell Immunol, 2009. **259**(1): p. 49-55.

23. Fujita, N., et al., *Involvement of Hsp90 in signaling and stability of 3-phosphoinositide-dependent kinase-1.* J Biol Chem, 2002. **277**(12): p. 10346-53.

24. Fukuyo, Y., et al., *Oxidative stress plays a critical role in inactivating mutant BRAF by geldanamycin derivatives.* Cancer Res, 2008. **68**(15): p. 6324-30.

25. Futami, M., et al., *RNAi-mediated silencing of p190Bcr-Abl inactivates Stat5 and cooperates with imatinib mesylate and 17-allylamino-17-demetoxygeldanamycin in selective killing of p190Bcr-Abl-expressing leukemia cells.* Leukemia, 2008. **22**(6): p. 1131-8.

26. Gano, J.J. and J.A. Simon, *A proteomic investigation of ligand-dependent HSP90 complexes reveals CHORDC1 as a novel ADP-dependent HSP90-interacting protein.* Mol Cell Proteomics, 2010. **9**(2): p. 255-70.

27. Garcia-Morales, P., et al., *Inhibition of Hsp90 function by ansamycins causes downregulation of cdc2 and cdc25c and G(2)/M arrest in glioblastoma cell lines.* Oncogene, 2007. **26**(51): p. 7185-93.

28. Germano, S., et al., *Geldanamycins trigger a novel Ron degradative pathway, hampering oncogenic signaling.* J Biol Chem, 2006. **281**(31): p. 21710-9.

29. Goes, F.S. and J. Martin, *Hsp90 chaperone complexes are required for the activity and stability of yeast protein kinases Mik1, Wee1 and Swe1.* Eur J Biochem, 2001. **268**(8): p. 2281-9.

30. Gould, C.M., et al., *The chaperones Hsp90 and Cdc37 mediate the maturation and stabilization of protein kinase C through a conserved PXXP motif in the C-terminal tail.* J Biol Chem, 2009. **284**(8): p. 4921-35.

31. Grbovic, O.M., et al., *V600E B-Raf requires the Hsp90 chaperone for stability and is degraded in response to Hsp90 inhibitors.* Proc Natl Acad Sci U S A, 2006. **103**(1): p. 57-62.

32. Hartson, S.D., et al., *Hsp90-mediated folding of the lymphoid cell kinase p56lck.* Biochemistry, 1996. **35**(41): p. 13451-9.

33. Hikri, E., S. Shpungin, and U. Nir, *Hsp90 and a tyrosine embedded in the Hsp90 recognition loop are required for the Fer tyrosine kinase activity.* Cell Signal, 2009. **21**(4): p. 588-96.

34. Hutchison, K.A., et al., *Reconstitution of the multiprotein complex of pp60src, hsp90, and p50 in a cell-free system.* J Biol Chem, 1992. **267**(5): p. 2902-8.

35. Imamura, T., et al., *Involvement of heat shock protein 90 in the degradation of mutant insulin receptors by the proteasome.* J Biol Chem, 1998. **273**(18): p. 11183-8.

36. Jaiswal, R.K., et al., *Nerve growth factor-mediated activation of the mitogen-activated protein (MAP) kinase cascade involves a signaling complex containing B-Raf and HSP90.* J Biol Chem, 1996. **271**(39): p. 23626-9.

37. Kawabe, M., et al., *Heat shock protein 90 inhibitor 17-dimethylaminoethylamino-17-demethoxygeldanamycin enhances EphA2+ tumor cell recognition by specific CD8+ T cells.* Cancer Res, 2009. **69**(17): p. 6995-7003.

38. Kudlicki, W., et al., *Identification of spectrin-related peptides associated with the reticulocyte heme-controlled alpha subunit of eukaryotic translational initiation factor 2 kinase and of Mr 95,000 peptide that appears to be the catalytic subunit.* J Biol Chem, 1987. **262**(20): p. 9695-701.

39. Lamphere, L., et al., *Interaction between Cdc37 and Cdk4 in human cells.* Oncogene, 1997. **14**(16): p. 1999-2004.

40. Lang, S.A., et al., *Targeting heat-shock protein 90 improves efficacy of rapamycin in a model of hepatocellular carcinoma in mice.* Hepatology, 2009. **49**(2): p. 523-32.

41. Lange, B.M., et al., *Cdc37 is essential for chromosome segregation and cytokinesis in higher eukaryotes.* EMBO J, 2002. **21**(20): p. 5364-74.

42. Lavictoire, S.J., et al., *Interaction of Hsp90 with the nascent form of the mutant epidermal growth factor receptor EGFRvIII.* J Biol Chem, 2003. **278**(7): p. 5292-9.

43. Lees-Miller, S.P. and C.W. Anderson, *Two human 90-kDa heat shock proteins are phosphorylated in vivo at conserved serines that are phosphorylated in vitro by casein kinase II.* J Biol Chem, 1989. **264**(5): p. 2431-7.

44. Lees-Miller, S.P. and C.W. Anderson, *The human double-stranded DNA-activated protein kinase phosphorylates the 90-kDa heat-shock protein, hsp90 alpha at two NH2-terminal threonine residues.* J Biol Chem, 1989. **264**(29): p. 17275-80.

45. Li, R., et al., *Hsp90 increases LIM kinase activity by promoting its homo-dimerization.* FASEB J, 2006. **20**(8): p. 1218-20.

46. Lipsich, L.A., J.R. Cutt, and J.S. Brugge, *Association of the transforming proteins of Rous, Fujinami, and Y73 avian sarcoma viruses with the same two cellular proteins.* Mol Cell Biol, 1982. **2**(7): p. 875-80.

47. Lochhead, P.A., et al., *A chaperone-dependent GSK3beta transitional intermediate mediates activation-loop autophosphorylation.* Mol Cell, 2006. **24**(4): p. 627-33.

48. Lowenberg, M., et al., *Glucocorticoids cause rapid dissociation of a T-cell-receptor-associated protein complex containing LCK and FYN.* EMBO Rep, 2006. **7**(10): p. 1023-9.

49. Matei, D., et al., *The platelet-derived growth factor receptor alpha is destabilized by geldanamycins in cancer cells.* J Biol Chem, 2007. **282**(1): p. 445-53.

50. Maulik, G., et al., *Modulation of the c-Met/hepatocyte growth factor pathway in small cell lung cancer.* Clin Cancer Res, 2002. **8**(2): p. 620-7.

51. McCleese, J.K., et al., *The novel HSP90 inhibitor STA-1474 exhibits biologic activity against osteosarcoma cell lines.* Int J Cancer, 2009. **125**(12): p. 2792-801.

52. Metchat, A., et al., *Mammalian heat shock factor 1 is essential for oocyte meiosis and directly regulates Hsp90alpha expression.* J Biol Chem, 2009. **284**(14): p. 9521-8.

53. Miyata, Y., et al., *Specific association of a set of molecular chaperones including HSP90 and Cdc37 with MOK, a member of the mitogen-activated protein kinase superfamily.* J Biol Chem, 2001. **276**(24): p. 21841-8.

54. Miyata, Y. and E. Nishida, *CK2 controls multiple protein kinases by phosphorylating a kinase-targeting molecular chaperone, Cdc37.* Mol Cell Biol, 2004. **24**(9): p. 4065-74.

55. Miyata, Y. and I. Yahara, *The 90-kDa heat shock protein, HSP90, binds and protects casein kinase II from self-aggregation and enhances its kinase activity.* J Biol Chem, 1992. **267**(10): p. 7042-7.

56. Miyata, Y. and I. Yahara, *Interaction between casein kinase II and the 90-kDa stress protein, HSP90.* Biochemistry, 1995. **34**(25): p. 8123-9.

57. Moran, D.M., et al., *Geldanamycin promotes premature mitotic entry and micronucleation in irradiated p53/p21 deficient colon carcinoma cells.* Oncogene, 2008. **27**(42): p. 5567-77.

58. Nair, S.C., et al., *A pathway of multi-chaperone interactions common to diverse regulatory proteins: estrogen receptor, Fes tyrosine kinase, heat shock transcription factor Hsf1, and the aryl hydrocarbon receptor.* Cell Stress Chaperones, 1996. **1**(4): p. 237-50.

59. Nakashima, T., et al., *New molecular and biological mechanism of antitumor activities of KW-2478, a novel nonansamycin heat shock protein 90 inhibitor, in multiple myeloma cells.* Clin Cancer Res, 2010. **16**(10): p. 2792-802.

60. Nieto-Miguel, T., et al., *Proapoptotic role of Hsp90 by its interaction with c-Jun N-terminal kinase in lipid rafts in edelfosine-mediated antileukemic therapy.* Oncogene, 2008. **27**(12): p. 1779-87.

61. Nimmanapalli, R., et al., *Regulation of 17-AAG-induced apoptosis: role of Bcl-2, Bcl-XL, and Bax downstream of 17-AAG-mediated down-regulation of Akt, Raf-1, and Src kinases.* Blood, 2003. **102**(1): p. 269-75.

62. Ochel, H.J., et al., *The benzoquinone ansamycin geldanamycin stimulates proteolytic degradation of focal adhesion kinase.* Mol Genet Metab, 1999. **66**(1): p. 24-30.

63. O'Keeffe, B., et al., *Requirement for a kinase-specific chaperone pathway in the production of a Cdk9/cyclin T1 heterodimer responsible for P-TEFb-mediated tat stimulation of HIV-1 transcription.* J Biol Chem, 2000. **275**(1): p. 279-87.

64. Ota, A., et al., *Specific regulation of noncanonical p38alpha activation by Hsp90-Cdc37 chaperone complex in cardiomyocyte.* Circ Res, 2010. **106**(8): p. 1404-12.

65. Park, J.H., et al., *Inhibitors of histone deacetylases induce tumor-selective cytotoxicity through modulating Aurora-A kinase.* J Mol Med, 2008. **86**(1): p. 117-28.

66. Prince, T., L. Sun, and R.L. Matts, *Cdk2: a genuine protein kinase client of Hsp90 and Cdc37.* Biochemistry, 2005. **44**(46): p. 15287-95.

67. Ramos, R.R., A.J. Swanson, and J. Bass, *Calreticulin and Hsp90 stabilize the human insulin receptor and promote its mobility in the endoplasmic reticulum.* Proc Natl Acad Sci U S A, 2007. **104**(25): p. 10470-5.

68. Rose, D.W., et al., *The 90-kilodalton peptide of the heme-regulated eIF-2 alpha kinase has sequence similarity with the 90-kilodalton heat shock protein.* Biochemistry, 1987. **26**(21): p. 6583-7.

69. Sawai, A., et al., *Inhibition of Hsp90 down-regulates mutant epidermal growth factor receptor (EGFR) expression and sensitizes EGFR mutant tumors to paclitaxel.* Cancer Res, 2008. **68**(2): p. 589-96.

70. Scholz, G.M., et al., *The molecular chaperone Hsp90 is required for signal transduction by wild-type Hck and maintenance of its constitutively active counterpart.* Cell Growth Differ, 2001. **12**(8): p. 409-17.

71. Schulte, T.W., et al., *Destabilization of Raf-1 by geldanamycin leads to disruption of the Raf-1-MEK-mitogen-activated protein kinase signalling pathway.* Mol Cell Biol, 1996. **16**(10): p. 5839-45.

72. Setalo, G., Jr., et al., *Estradiol-induced phosphorylation of ERK1/2 in explants of the mouse cerebral cortex: the roles of heat shock protein 90 (Hsp90) and MEK2.* J Neurobiol, 2002. **50**(1): p. 1-12.

73. Shang, L. and T.B. Tomasi, *The heat shock protein 90-CDC37 chaperone complex is required for signaling by types I and II interferons.* J Biol Chem, 2006. **281**(4): p. 1876-84.

74. Shimamura, T., et al., *Epidermal growth factor receptors harboring kinase domain mutations associate with the heat shock protein 90 chaperone and are destabilized following exposure to geldanamycins.* Cancer Res, 2005. **65**(14): p. 6401-8.

75. Suttitanamongkol, S., R. Polanowska-Grabowska, and A.R. Gear, *Heat-shock protein 90 complexes in resting and thrombin-activated platelets.* Biochem Biophys Res Commun, 2002. **297**(1): p. 129-33.

76. Taherian, A., P.H. Krone, and N. Ovsenek, *A comparison of Hsp90alpha and Hsp90beta interactions with cochaperones and substrates.* Biochem Cell Biol, 2008. **86**(1): p. 37-45.

77. Takata, Y., et al., *Functional importance of heat shock protein 90 associated with insulin receptor on insulin-stimulated mitogenesis.* Biochem Biophys Res Commun, 1997. **237**(2): p. 345-7.

78. Trentin, L., et al., *Geldanamycin-induced Lyn dissociation from aberrant Hsp90-stabilized cytosolic complex is an early event in apoptotic mechanisms in B-chronic lymphocytic leukemia.* Blood, 2008. **112**(12): p. 4665-74.

79. Tse, A.N., et al., *90-kDa heat shock protein inhibition abrogates the topoisomerase I poison-induced G2/M checkpoint in p53-null tumor cells by depleting Chk1 and Wee1.* Mol Pharmacol, 2009. **75**(1): p. 124-33.

80. Uma, S., et al., *Hsp90 is obligatory for the heme-regulated eIF-2alpha kinase to acquire and maintain an activable conformation.* J Biol Chem, 1997. **272**(17): p. 11648-56.

81. Wang, Y., et al., *Cotreatment with panobinostat and JAK2 inhibitor TG101209 attenuates JAK2V617F levels and signaling and exerts synergistic cytotoxic effects against human myeloproliferative neoplastic cells.* Blood, 2009. **114**(24): p. 5024-33.

82. Webb, C.P., et al., *The geldanamycins are potent inhibitors of the hepatocyte growth factor/scatter factor-met-urokinase plasminogen activator-plasmin proteolytic network.* Cancer Res, 2000. **60**(2): p. 342-9.

83. Wrighton, K.H., X. Lin, and X.H. Feng, *Critical regulation of TGFbeta signaling by Hsp90.* Proc Natl Acad Sci U S A, 2008. **105**(27): p. 9244-9.

84. Xie, Q., et al., *Geldanamycins exquisitely inhibit HGF/SF-mediated tumor cell invasion.* Oncogene, 2005. **24**(23): p. 3697-707.

85. Yang, K., et al., *Hsp90 regulates activation of interferon regulatory factor 3 and TBK-1 stabilization in Sendai virus-infected cells.* Mol Biol Cell, 2006. **17**(3): p. 1461-71.

86. Yang, W., et al., *Interaction of activated Cdc42-associated tyrosine kinase ACK2 with HSP90.* Biochem J, 2004. **382**(Pt 1): p. 199-204.

87. Yorgin, P.D., et al., *Effects of geldanamycin, a heat-shock protein 90-binding agent, on T cell function and T cell nonreceptor protein tyrosine kinases.* J Immunol, 2000. **164**(6): p. 2915-23.

88. Yun, B.G. and R.L. Matts, *Differential effects of Hsp90 inhibition on protein kinases regulating signal transduction pathways required for myoblast differentiation.* Exp Cell Res, 2005. **307**(1): p. 212-23.

89. Yun, C.H., et al., *Geldanamycin inhibits TGF-beta signaling through induction of Hsp70.* Arch Biochem Biophys, 2010. **495**(1): p. 8-13.

90. Zhang, H., et al., *Hsp90/p50cdc37 is required for mixed-lineage kinase (MLK) 3 signaling.* J Biol Chem, 2004. **279**(19): p. 19457-63.

91. Ziemiecki, A., et al., *Association of the heat shock protein hsp90 with steroid hormone receptors and tyrosine kinase oncogene products.* Biochem Biophys Res Commun, 1986. **138**(3): p. 1298-307.

1. Hartson, S.D. and R.L. Matts, *Association of Hsp90 with cellular Src-family kinases in a cell-free system correlates with altered kinase structure and function*. Biochemistry, 1994. **33**(30): p. 8912-20.

93. Ramensky V et al., *Human non-synonymous SNPs: server and survey.* Nucleic Acids Res*,* 2002. **30**(17): 3894-900

94. Schwarz J.M. Et al., ***MutationTaster evaluates disease causing potential of sequence alterations.*** Nat Methods, 2010. **7**(8): p. 575-6.

95. Peri, S. et al., *Development of human protein reference database as an initial platform for approaching systems biology in humans*. Genome Research, 2003. **13**: p. 2363-2371.

96. Prasad, T. S. K. et al., *Human Protein Reference Database - 2009 update*. Nucleic Acids Research, 2009. **37**: D767-D772.

97. Stark C. et al., ***BioGRID: a general repository for interaction datasets.*** Nucleic Acids Res, 2006. **34**: D535-9.

98. Breitkreutz B.J. et al., ***The BioGRID Interaction Database: 2008 update.*** Nucleic Acids Res, 2008. 36: D637-40.

99. Savitski M.M. et al., *Targeted data acquisition for improved reproducibility and robustness of proteomic mass spectrometry assays.* J Am Soc Mass Spectrom., 2010. **21**(10): p. 1668-79. Epub 2010 Jan 25.
