# Supplementary material for: Hsp90 inhibition differentially destabilises MAP kinase and TGF-beta signalling components in cancer cells revealed by kinase-targeted chemoproteomics
Source: BMC Cancer. 2012 Jan 25;12:38. doi: 10.1186/1471-2407-12-38 (PMC3342885; doi:10.1186/1471-2407-12-38)
Supplement: Additional file 8 — Supplementary_Documents_Description. Description of each supplementary table content and supplementary figure description. [file 1471-2407-12-38-S8.DOC]

**Supplementary Tables 1-6 and Supplementary Figure 1:**

**Supplementary Table 1:** Relative quantification of kinases identified from Hs68, SW480, U2OS and A549 cells treated with geldanamycin for 12 or 24h following enrichment on Kinobeads (from two independent experiments). Fold change is the ratio between geldanamycin treated and DMSO treated cells.

**Supplementary Table 2:** Table summarizes quantification data and indicates known clients including references for interactions and Hsp90 inhibitor effects. Known Hsp90 clients were identified using the list of Hsp90 interactors of the Picard lab (www.picard.ch), HPRD ([www.hprd.org](http://www.hprd.org/), Supplementary Ref. 95, 96) and BioGRID ([www.thebiogrid.org](http://www.thebiogrid.org/), Supplementary Ref. 97, 98) databases, the publication of Citri et al. (Supplementary Ref. 14) and literature search. Criterion for classification as a substrate was a reported Hsp90 inhibition effect. If contradictory data was found, the kinase was considered a known client. Potential new clients are listed according to a high or low confidence (as classified in the main text).

**Supplementary Table 3:** Relative quantification of Hsp90 chaperone protein complex identified from Hs68, SW480, U2OS and A549 cells treated with geldanamycin for 24h. Fold change is the ratio between geldanamycin treated and DMSO treated cells. Quantification was performed directly from lysate from two independent experiments.

**Supplementary Table 4:** Kinetics of individual kinases in all four cell lines. Cells were treated with geldanamycin for 12 or 24h. Fold change is the ratio between geldanamycin treated and DMSO treated cells. Thresholds to determine kinetics patterns were log2 fold changes of 0.4, -0.4 and -0.8.

**Supplementary Table 5:** Kinases showing differential quantification when their levels are compared to those from HS68 following 24 h treatment with geldanamycin. Fold change is the ratio between geldanamycin treated and DMSO treated cells. Figures are the difference in log2 fold change between each cancer cell line and HS68.

**Supplementary Table 6:** Relative quantification of kinases identified from Hs68 and SW480 cells treated with geldanamycin for 24h with or without MG132 following enrichment on Kinobeads (from two independent experiments). Fold change is the ratio between different treatments.

**Supplementary Table 7:**

Results of the sequencing of 144 kinase genes in Hs68, SW480, U2OS and A549 cells. Shown are missense mutations. Highlighted in yellow are mutations that are not annotated in dbSNP and are predicted to have an impaired protein function by using Polyphen and MutationTaster algorithms.

**Supplementary Figure 1:**

Shown are the western blot results of two independent experiments comparing DMSO and geldanamycin treatment for 24h. Western blotting results were assigned a trend of up- or downregulation or no change and compared to the trend of MS. In total we obtained data for 24 of the kinases quantified in the MS. Adding up the data from four cell lines we obtained a total of 60 measurements for the 24 kinases. For 17 of these we did not find a consistent response to GA treatment in both experiments of WB. . Of the remaining 43 measurements 32 (74%) confirmed the trend observed in the MS. Among these are known client proteins like Cdk1, Cdk2, Cdk7, Lyn and RSK1. This supports the validity of our results. For 11 measurements (26%) we did not observe the same trend after Hsp90 inhibition as in the MS.
